# Adenocarcinoma Ex Goblet Cell Carcinoid of Appendix: Two Case Reports

**DOI:** 10.1155/2017/5930978

**Published:** 2017-08-14

**Authors:** Yu-Ting Wang, Yi-Ru Li, Tuan-Ying Ke

**Affiliations:** Department of Pathology, Chung Shan Medical University Hospital, No. 110, Sec. 1, Jianguo N. Rd., Taichung City 40201, Taiwan

## Abstract

Adenocarcinoma ex goblet cell carcinoid is a rare tumor incidentally found in specimens of appendicitis. Most patients present with acute abdomen, similar to acute appendicitis. Here we present two cases, which were found incidentally after operation. We give a brief summary about clinical and biological behavior of this entity.

## 1. Introduction

Goblet cell carcinoid is a rare tumor and was first described by Gagne in 1969. It is an uncommon primary tumor of the appendix characterized by dual neuroendocrine and glandular differentiation. It has distinct biological behavior and is different from mucinous neoplasm, appendiceal carcinoid, or adenocarcinoma originating from the appendix. Here, we present two cases. Case one was misinterpreted as a carcinoma favoring gastrointestinal origin at first due to the following two reasons:

- Failure of immunohistochemical confirmation about neuroendocrine differentiation in areas of goblet cell crypt pattern
- The presence of a large amount of tumor cells within mucin pools that mimic signet-ring cells and neoplastic intestinal cells.

Case two presented with a typical morphological and immunohistochemical pattern. Here, we discuss the clinical presentation, morphological patterns, and immunohistochemical patterns and give a paper review of the biological characteristics of the entity.

## 2. Case 1

**Figure 1:**
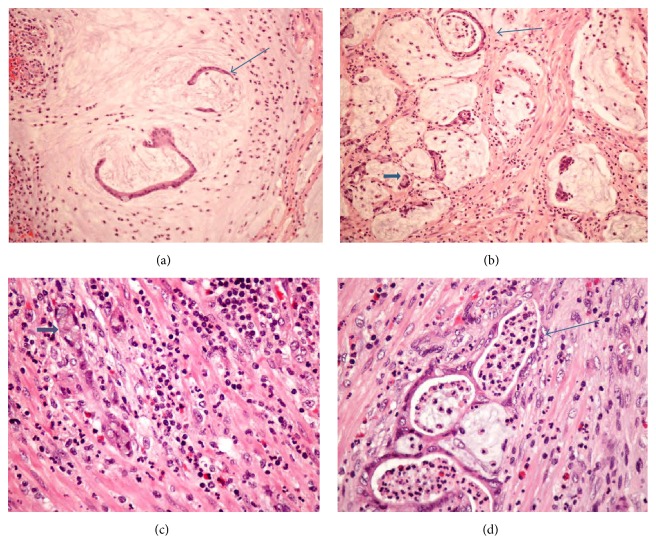
(a) Case 1: intestinal-like cells (labelled as thin arrow) floating in mucin pools. (hematoxylin-eosin, original magnifications ×200). (b) Case 1: intestinal-like cells (labelled as thin arrow) and signet-ring cell crypt (labelled as broad arrow) in mucin pools (hematoxylin-eosin, original magnifications ×200). (c) Case 2: areas of goblet crypt-like cells (labelled as broad arrow) (hematoxylin-eosin, original magnifications ×400). (d) Case 2: intestinal-like cells (labelled as thin arrow) (hematoxylin-eosin, original magnifications ×400).

A 49-year-old male presented with acute abdominal pain for one day. CT finding showed dilated appendix due to enhanced thickened mucosa and sign of fat stranding adjacent to soft tissue. Based on the impression of acute appendicitis, an appendectomy was performed. Pathologically, there was mucin pool formation with foci of morphologically bland neoplastic intestinal cells (Figure 1(a)) to poorly differentiated hyperchromatic cells with focal signet-ring-like cells (Figure 1(b)) in the submucosa and muscular layer with surgical margins showing the presence of tumor cells. Extensive lymphovascular invasion and neural invasion were seen. Immunohistochemical results of CD56 and synaptophysin were very weak in the appendix. Because the immunohistochemical results failed to confirm goblet cell carcinoid, we signed out the pathological report with carcinoma, and we suggested that the clinicians check the gastrointestinal tract to rule out any gastrointestinal carcinoma with appendiceal metastasis. After a complete check-up, no other gastrointestinal lesions were found but a primary pulmonary adenocarcinoma was found (confirmed by immunohistochemical results of TTF-1 positive, chromogranin A negative, synaptophysin negative, and napsin A positive for tumor cells). Complete right hemicolectomy was performed. Residual tumors were found on the muscular wall of the cecum and the serosal layer of the appendiceal stump (pT4aN0). No regional lymph node metastasis was seen. Morphologically, the residual tumor was similar to the previous tumor but scanty mucin pool formation and more foci of goblet crypt carcinoid were found. Immunohistochemical results showed CEA(+), CK7(−), synaptophysin(+), CD56(+), chromogranin A(+), and Ki-67 (40%). So, adenocarcinoma ex goblet cell carcinoid was the final diagnosis. After a clinical follow-up of one year, no tumor recurrence was found.

## 3. Case 2

A 64-year-old male presented with acute abdominal pain for 2 days. KUB film showed fecal material and bowel gas in the gastrointestinal tract. Pathologically, focal signet-ring-like cells admixed with goblet crypt-like cells in the submucosal layer to the subserosal layer were present (Figures 1(c) and 1(d)). Prominent perineural invasion and lymphovascular invasion were seen. Immunohistochemical profiles demonstrated dual differentiation: CK7(−), CK20(+), CDX2(+), CD56(+), synaptophysin (+), and chromogranin (+) in the tumor cells. Clinical examination revealed no tumors in other areas. Complete right hemicolectomy also showed no residual tumor. Adenocarcinoma ex goblet cell carcinoid was the final diagnosis. After a clinical follow-up of one year, no tumor recurrence was found.

## 4. Discussion

Goblet cell carcinoid, first described in 1969 by Gagne et al., is a very rare tumor occurred in appendix [1]. It is generally found incidentally on the impression of appendicitis. The tumor is usually derived from the deep lamina propria of the appendix, with contiguous spread to the submucosal layer and muscular layer, forming concentric thickening of appendiceal wall and then resulting in acute appendicitis.

It is believed that the tumor originates from pluripotent intestinal crypt base stem cells with dual differentiation by presence of mucin droplet and neuroendocrine secretory granules found in tumor cells [2].

Goblet cell carcinoid usually displays several histological pattern [3]. Our case 1 showed three different patterns, and case 2 showed two different patterns. Reid et al. reviewed 77 cases, they also found that none of the cases had a pure histologic pattern, and in any given case there was a mixture of at least two or more of these patterns [3]. They mentioned the pattern that stromal mucin formation admixed with neoplastic intestinal cells. Ng et al. found coexistence of goblet cell carcinoid and mucinous neoplasm and thought common tumor stem cell with potential of multiple lineage differentiations resulted in different histological patterns [4]. Are they downstream mutations from goblet cell carcinoid or concurrent different pathway from stem cells? We suggested more studies to confirm the theory.

What is the clinical significance of these different histological patterns? Tang et al. classified goblet cell carcinoid in three subgroups [5]: pure goblet cell carcinoid, adenocarcinoma ex goblet cell carcinoid (signet-ring cell type), and adenocarcinoma ex goblet cell carcinoid (poorly differentiated type) and they found, although patients presented with TNM stage IV, pure goblet cell carcinoid still has very good survival rate, while poorly differentiated type and signet-ring cell type show poor prognosis. Taggert et al. found the prognosis of goblet cell carcinoid is negatively correlated with proportion of carcinomatous components [1]. And extra-appendiceal spread, margin positivity, and disease stage show positive correlation with prognosis.

In clinical practice, it is important to aware these different histological patterns and total appendix should be submitted to find the different patterns that would denote prognosis. Because immunohistochemical result of neuroendocrine markers would be negative or only patch staining [2], especially in cases with more adenocarcinomatous component [1], we suggested neuroendocrine marker should stain more than one slide of the tumor, and the focal patch staining pattern should be considered positive for the entity. In addition, goblet cell carcinoid ex adenocarcinoma usually presented with a coelomic peritoneal spreading pattern, especially in late stage; we suggested if tumors with goblet cell crypt-like pattern were found pathologically in the abdominal cavity, clinically presenting with coelomic dissemination, rather than solid organ metastasis, differential diagnosis of the entity may be included and clinical intervention of appendix should be done.

For early stage (tumor confined the submucosa), appendectomy alone is adequate. If tumor is found in margin of specimen of appendectomy or tumor in advanced stage, right hemicolectomy is suggested. For female patients, because of the high possibility of bilateral ovarian involvement, prophylactic bilateral salpingo-oophorectomy is suggested especially in postmenopausal women [3]. For the clinician, after surgical resection, clinical follow-up is suggested.

To sum up, goblet cell carcinoid is a tumor different from appendiceal carcinoma. It displays different morphological components and may be prognostically important, so the proportion of different tumor components should be mentioned. Presence of characteristic goblet cell crypt-like cells is a good key to diagnosis. Immunohistochemical stain of neuroendocrine marker is always variable and shows focal patch pattern. The tumor usually behaves by forming peritoneal surface spread and lymph node metastasis, rather than solid organ involvement and hematological spread. For the clinician, high propensity for gynecological tract involvement should be concerned for female patients. Surgical option is based on disease stage and margin status. Long-term follow-up for recurrence is suggested.
